# Are they publishing? A descriptive cross-sectional profile and bibliometric analysis of the journal publication productivity of Italian physiotherapists

**DOI:** 10.1186/s40945-017-0042-8

**Published:** 2018-01-02

**Authors:** Stefano Vercelli, Elisa Ravizzotti, Matteo Paci

**Affiliations:** 1 Laboratory of Ergonomics and Musculoskeletal Disorders Assessment, Division of Physical Medicine and Rehabilitation, Istituti Clinici Scientifici Maugeri SpA-SB, Via per Revislate 13, I-28010 Veruno, NO Italy; 2 Master of Science in Rehabilitation Sciences of the Health Professions, University of Genova, Varese, Italy; 3 Unit of Functional Recovery, Azienda USL Toscana Centro, Area Prato, Italy

**Keywords:** Bibliometrics, Authorship, Scientific output indicators

## Abstract

**Background:**

In a clinical science-based profession such as physiotherapy, research is mandatory to update knowledge and to provide cost-effective, high quality treatments. This study aimed to provide point prevalence of Italian physiotherapists who are academics, holding a PhD degree, or being authors of scientific papers. The scientific journal productivity of physiotherapists was also thoroughly analyzed.

**Methods:**

A descriptive cross-sectional study was carried out on all Italian physiotherapists. Academics, postdoctoral research fellows, and PhD graduates were identified by searching the Italian Ministry of Education, University and Research (MIUR), Italian Society of Physiotherapy, and university websites. Then, authors of articles indexed in Scopus were searched. The following data were extracted: type of affiliation, authorship order, H-index, number of publications and citations, name of journals, year of publication, and journal’s Impact Factor.

**Results:**

The prevalence of academics, physiotherapists holding a PhD, or being author was 0.01%, 0.05%, and 0.56%, respectively. We identified 1083 papers co-authored by Italian physiotherapists, and their number has progressively increased over the years (*p* < 0.001). There was a significant difference between researchers and clinicians in both publication productivity (*p* < 0.01), citations (p < 0.01), and H-Index (*p* = 0.05). Articles were published in 359 different journals, receiving a total of 13,373 citations.

**Conclusions:**

Despite the low prevalence of faculty members and the reduced availability of PhD programs in Italy (forcing some students to study abroad), the quantity and quality of journal productivity is growing fast, and an increasing number of physiotherapists are involved in research activities.

## Background

The credibility and professional development of physiotherapy is growing fast, thanks to the scientific knowledge produced by their members and the clinical application of proven research findings [1]. In a healthcare system that requires treatment to be of high quality and cost effective, research is mandatory to validate current practice and guide the development of clinical guidelines. To transfer knowledge to practice and obtain the expected impact, the results of research must be documented in publications in order to have them validated and made legitimate for use [2].

In a previous study [3], the scientific productivity of Italian physiotherapists was analyzed. Results indicated a point prevalence of authors in 2012 of about 0.34% - with authors being prevalently from the northern and central regions of Italy (Tuscany, Emilia Romagna and Piedmont) - and a steady increase in the number of published articles over years. The authors were working most frequently in non-scientific institutes (67%), but those working in research institutes had higher bibliometric indicators. The academic professional profile of authors was not fully explored, because at that time there was only one physiotherapist working in a full-time academic position. However, in the last few years substantial changes have taken place in physiotherapy education in Italy. The Master’s level degree (the ‘*Laurea Magistrale’*) was introduced in late 2004, and the first physiotherapists graduated in 2008. Since then, a few programs have begun offering the Doctor of Philosophy (PhD) degree to physiotherapists in our country. Moreover, in 2012 the Italian Ministry of Education, University and Research (Ministero dell’Istruzione, dell’Università e della Ricerca, MIUR) introduced a new process for the appointment of professors, which has been described elsewhere [4]. These substantive changes have prompted the need to determine more precisely the profile of Italian physiotherapists involved in research activities, and to provide quantitative indicators of their productivity.

The primary aim of this study was to provide point prevalence of Italian physiotherapists who: a) have university roles; b) holding a PhD degree; and c) have written at least one article published in peer-review journals indexed in the Scopus database. The secondary objectives were to describe the profile of academics and educational programs, and to analyze the scientific journal productivity of authors.

## Methods

This was a descriptive cross-sectional study.

The study population was represented by all Italian physiotherapists with a bachelor’s degree (3 years), working either in Italy or abroad. Foreign professionals working in Italy were excluded.

The number of Italian professionals was recently estimated by the MIUR and the Italian Association of Physiotherapists (AIFI) in a survey conducted to establish the national educational needs [5]. In this study, the data provided by the ministerial survey was used to calculate prevalence.

To identify those who have university roles - i.e. professors and postdoctoral research fellows - the MIUR website [6] was searched. The academic section area (*Settore Scientifico Disciplinare*, SSD) tag was used to retrieve positions in the sector “Sciences of nursing, rehabilitation and neuropsychiatric techniques” (MED/48). The MED/48 filter option was selected, and academics were classified as: full professors, associate professors, and researchers. The curriculum of each person was then carefully analyzed to identify those who were physiotherapists. The same strategy was used to find postdoctoral research fellows and the university educational programs (bachelor, graduate, and PhD) offered in Italy.

To find physiotherapists holding a PhD, three strategies were used. First, all PhD programs offered to physiotherapists in the last 5 years were extracted from the MIUR database. Each PhD program’s website - if available - was then searched to find information on students and former students, and physiotherapists were identified. Second, the list of PhDs and PhD students available on the Italian Society of Physiotherapy (SIF) website was downloaded [7]. Third, each PhD or PhD student identified was contacted to find any other colleagues or fellow students.

Italian physiotherapists who published in peer-review journals indexed in the Scopus database were then searched with a previously described strategy [3].

For each author the following data were extracted: type of work based on affiliation (academics - i.e. professors and researchers - and those working in scientific institutes such as the *Istituti di Ricovero e Cura a Carattere Scientifico*, were termed ‘researchers’, while those working in hospitals, hospices, retirement homes, or professional studies were termed ‘clinicians’), total number of publications per author, number of citations received per author, authorship order (first, second, and last position were considered as ‘most relevant’ in multi-authored articles, potentially reflecting a greater contribution from those authors than of collaborators in other positions), and H-index. The H-index (i.e. the number of articles published by the researcher which obtained a citation number greater than or equal to the number of articles) was proposed in 2005 to quantify the productivity and the impact of researchers, and it is used also by the MIUR to evaluate the activity of researchers.

After deleting duplicates generated by authors’ collaboration, all articles published in journals indexed in Scopus were then identified, excluding conference papers and book chapters. No limitation on publication period or language was set. For each publication, the following information were extracted: name of journal, year of publication, total number of citations received, and journal’s Impact Factor (IF). The IF is a measure reflecting the yearly average number of citations of recent articles published in that journal. In this study, the Web of Science 2015 IF values were used.

Counts, percentages, and ratios were used to summarize the data. A Mann-Whitney U test was used to analyze differences between researchers and clinicians in H-Index, number of publications and citations received per author. The correlation between year and number of published articles was estimated by the Spearman rank-order correlation coefficient (rho). For all tests, the level of statistical significance was set at 0.05.

## Results

In 2017, the Italian population of physiotherapists was estimated at about 65,000 professionals [5].

To date, 7 physiotherapists - 1 full professor, 1 full temporary professor, and 5 researchers - are listed in the MED/48 section area of Italian university faculty members, wich resulted in a prevalence of 0.01%. The full professor was appointed in 2005, and the associate professor in late 2016. Besides the academics, the MIUR website currently lists 4 postdoctoral research fellows among physiotherapists.

A total of 85 bachelor (undergraduate) programs of physiotherapy are currently being taught in Italy, to which must be added 16 master (graduate) programs and 5 doctorate programs (XXXII cycle, year 2016) accessible to physiotherapists. Thirty-one Italian physiotherapists holding a PhD have been identified (half of whom gained the title in the last 18 months), with a prevalence of 0.05%. At least 14 more physiotherapists are currently in training, either in Italy or abroad (Belgium, Great Britain, Denmark, Netherlands, and Spain).

**Fig. 1:**
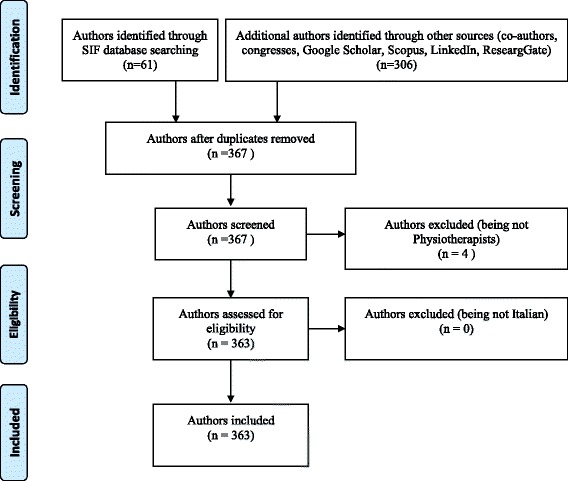
Flow chart of authors selection process

Up to 2016, 363 Italian physiotherapists were identified as having been involved in scientific journal publication (Fig. 1), wich resulted in a prevalence of 0.56%. Of all authors, 242 (67%) were researchers and 121 (33%) were clinicians.

Overall, they appeared as authors 1820 times and, after deleting duplicates generated by collaborations, they produced a total of 1083 different articles published in journals indexed in the Scopus database. The distribution of H-Index, number of articles published, and number of citations received by researchers and clinicians are shown in Table 1. There was a significant difference in both publication productivity (*p* < 0.01), citations received (*p* < 0.01), and H-Index (*p* = 0.05) between researchers and clinicians.

**Table 1:** Distribution of articles published and citations received by each author, and H-Index distribution for authors

	All authors *N* = 363	Researchers *N* = 242	Clinicians *N* = 121
Articles published per author			
Cumulative	1820	1482	338
Mean	5	6.1	2.8
Interquartile Range	1–4	1–6	1–3
Min-Max	1–53	1–53	1–32
Citations per author			
Cumulative	21,063	16,551	4512
Mean	58	68.4	37.3
Interquartile Range	2–48	3–54.5	2–36
Min-Max	0–898	0–898	0–397
H-Index			
Cumulative	809	600	208
Mean	2.2	2.5	1.7
Interquartile Range	1–3	1–3	1–2
Min-Max	0–16	0–16	0–11
% H-Index >2	17%	29%	12%

In the sequences of authors, Italian physiotherapists were listed as authors in first, second, or last position (i.e. potentially reflecting higher contribution credit) 54% of times.

**Fig. 2:**
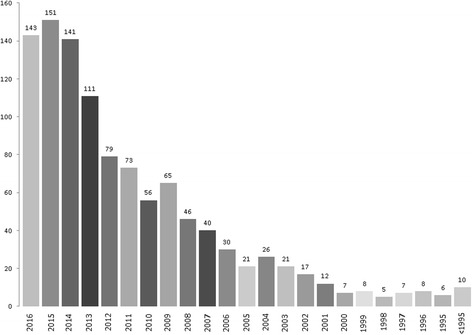
Number of published articles per year

The number of articles published has increased steadily over the years (rho = 0.977, *p* < 0.001) (Fig. 2).

The 1083 papers identified received a total of 13,373 citations. In 74% of the articles accounting for 71% of citations researches featured as authors; in 16% of the articles accounting for 21% of citations clinicians featured, while in 10% of articles receiving 8% of the total citations the authors represented a collaboration between the two (Table 2).

**Table 2:** Distribution of citations per article among author affiliation types

	All authors	Only Researchers	Only Clinicians	Researchers + Clinicians
Articles authored	1083	801	169	113
Citations per article				
Cumulative	13,373	9466	2847	1060
Mean	12.3	11.8	16.8	9.4
Interquartile Range	1–14	0–13	1–23	1–12
Min-Max	0–194	0–194	0–182	0–52

Overall, 359 different journals were identified, only one-third of them being listed in the Web of Science Rehabilitation category; 252 journals had an IF, and 80% of all articles were published in these journals (Table 3).

**Table 3:** Counts of journals and articles published with their IF distribution

	Number
Journals	
All	359
Journals with IF	252
Included in WOS Rehabilitation Category	113
Articles published	
All	1083
in Journals with IF	844
Mean Journal IF	2.81
Interquartile Range Journal IF	1.839–3.057
Min-Max Journal IF	0.075–44.002

Legend: *WOS* Web of Science, *IF* impact factor

## Discussion

Conducting research and publishing in scientific journals is regarded as a mandate for researchers, especially those working within universities. The primary purpose of this study was to calculate point prevalence of Italian physiotherapists who are academics, holding a PhD degree, or being authors of scientific papers.

The very low prevalence (0.01%) of physiotherapists who are professors or academic researchers in Italy has already been described in the recent past [4, 8]. However, since 2012 the procedure of appointing new professors in Italy has changed and in April 2017 seven Italian physiotherapists were qualified by a national ministerial committee and may be called upon in the future to fill an academic position [9].

Italian physiotherapists holding a Doctoral degree are about 0.05%. This prevalence is still very low, but it is in line with emerging countries such as Brazil [10] and it is expected to grow very rapidly within the next few years, with many PhD students currently in training. Some of them have chosen to continue their studies abroad, because of the few existing PhD programs in Italy. This highlights the intention of Italian physiotherapists to seek higher education and the urgent need to expand the number of research fellowships in this area.

Despite the small number of academics and PhDs in physiotherapy in Italy, the national scientific output in the rehabilitation area has achieved important bibliometric indicators worldwide: in the SCImago Country rank for number of published articles Italy was 9th, with 301 papers in 2016 and 4183 in the decade 1996–2016 [11]. Of note, not all of these articles were co-authored by physiotherapists, and not all of the authors were academics.

In this study, more than 1000 articles were found to be co-authored by at least 363 physiotherapists, who most often had a scientific affiliation. In a previous review [3], 139 authors producing 517 articles were identified between 1993 and 2012. In the last 5 years, the prevalence of authors passed from 0.34% to 0.56%, and both article production and number of authors has doubled. In the same period, the percentage of those affiliated with universities or research institutes versus non-scientific institutes has reversed. Academic or scientific institute affiliations could facilitate initiatives and opportunities, establishing contacts between colleagues, producing new publications and sharing knowledge. Besides their own scientific activity, senior researchers and professors also play an important role as mentors, serving as a catalyst for young researchers and promoting their development. However, it should be noted that they rarely are full-time researchers (as is the case in other countries) but are more often employed with a combined (or even prevalently) clinical role. Researchers had a significantly higher H-index (*p* = 0.05), they were the profile most often involved in publishing (*p* < 0.01) and they received more citations (*p* < 0.01) than clinicians. While it was not surprising that researchers had a greater scientific output (Table 1), it was surprising that the articles that received a higher average number of citations were those published by clinicians (Table 2). This may be explained by different reasons. One may be that researchers have published many more articles, and especially the recent ones not yet cited may have reduced the mean citations number. For example, one of the researchers published 40 articles that received 341 total citations. Not considering the 15 articles that have been written in the last 2 years and which have received 13 citations together, the average citations per article of that author would go from 8.5 to 13. On the other hand, this demonstrates also the good quality of the articles written by clinicians. Over and above the scholarly expectations of the academic milieu, the development of a profession depends also on its members, working to refine and expand the body of knowledge that guides practice in that area. Although clinical physiotherapists generally devote little time to research activities, they are in a better place to carry out clinical trials, clinical auditing, case studies, and single-subject experimental designs [12].

Criteria for authorship have been discussed at length, but a simple way to determine credit associated with the sequence of authors’ names is still missing [13]. In multi-authored papers, the first author position is traditionally assigned to the person who makes the greatest contribution, and the subsequent order of authors reflects the descending importance of their contribution. However, evaluation committees and funding bodies often consider last authorship as a sign of successful group leadership and make this a criterion in hiring, granting, and promotion [13]. In this study, higher credit was arbitrarily assigned to the first, second, and last author’s position. Even in multi-authored, multidisciplinary joint papers, Italian physiotherapists were ranked in one of these positions more than half of the time, potentially reflecting an important contribution to authorship.

Since the first publication co-authored by an Italian physiotherapist appeared in 1981 [14], the number of articles published per year has steadily increased (Fig. 1), with more than 50% of all publications produced between 2012 and 2016. However, it should be noted that a small group of highly productive physiotherapists (about 25) were responsible for more than 20% of all publications. Articles were published in 359 different journals being indexed in Scopus, of which 252 had an IF. The mean IF of these journals was 2.81 (ranging from 0.075 to 44.002, interquartile range [IQR] 1.839–3.057). In 2015, the mean IF of the 113 journals indexed in the Web of Science Rehabilitation category was 1.324 (ranging from 0.094 to 4.035, IQR 0.73–1.711). These data demonstrate the high average quality of the scientific production of Italian physiotherapists, who have published a significant proportion of their studies in top-level international journals.

Journal productivity is a complex issue reaching beyond the existence of research degree programs [1], and the results of this study demonstrate that good levels of publication productivity may be reached regardless of the insufficient number of academic figures, institutions, or leadership of the programs. In place of university, the function of promoting culture and communication with professional networks, research emphasis, assertive-participative governance, mentoring and motivation, is currently carried out by scientific societies and associative scientific groups. However, we hope that in the near future the number of faculty members and PhD fellows will be further increased in Italy, and that journals supported by scientific societies - such as the *Archives of Physiotherapy* - will get Scopus indexing. This could finally promote development of physiotherapy leadership and governance of university programs.

The conclusions that we can draw from this investigation may be limited by some factors. First, the approach used to find authors was not exhaustive because no database provides search options by both profession and country. Second, only the journals indexed in the Scopus database were considered. Although this choice may have underestimated the results, we believe that it was justified since Scopus provides a strong coverage of the physiotherapy literature [15] and it is currently used by the MIUR to evaluate researcher’s journal production. Then, internet sites were used to find academics and university programs instead of directly contacting the MIUR or universities. Therefore, the accuracy of information depends on the state of updating their websites. Moreover, publication productivity was measured by standardized bibliographic indicators based on number of articles and citations received, such as H-index and IF. We recognize that there are other forms of publications and indexes, and that numerous criticisms have been made about the use of these particular parameters [16]. However, this choice was done because of the availability of information and their widespread use to analyze the impact of publications on the scientific community [17] and to monitor scientific productivity of physiotherapists in other countries [1, 2, 10].

## Conclusions

This study presented an updated point prevalence of Italian physiotherapists who are academics, holding a PhD degree, or being authors of scientific papers. The scientific journal productivity of physiotherapists was also thoroughly analyzed. Results indicate that faculty members among physiotherapists are still very low, and there are insufficient PhD programs to meet the demand. However, the quantity and quality of journal publication productivity is growing fast, with an increasing number of physiotherapists involved in research activities.

## Abbreviations

IF: Impact factor
PhD: Doctor of Philosophy degree
